# The effects of an integrated care intervention for the frail elderly on informal caregivers: a quasi-experimental study

**DOI:** 10.1186/1471-2318-14-58

**Published:** 2014-05-01

**Authors:** Benjamin Janse, Robbert Huijsman, Ruben Dennis Maurice de Kuyper, Isabelle Natalina Fabbricotti

**Affiliations:** 1 Erasmus University Rotterdam, Institute of Health Policy and Management, P.O. Box 1738, Rotterdam, DR 3000, The Netherlands; 2 Huisartsenpraktijk Arnemuiden, Prins Bernhardstraat 2, Arnemuiden, EZ4341, The Netherlands

**Keywords:** Integrated care, Frail elderly, Informal caregiver, Perceived health, Objective burden, Subjective burden, Quality of life

## Abstract

**Background:**

This study explored the effects of an integrated care model aimed at the frail elderly on the perceived health, objective burden, subjective burden and quality of life of informal caregivers.

**Methods:**

A quasi-experimental design with before/after measurement (with questionnaires) and a control group was used. The analysis encompassed within and between groups analyses and regression analyses with baseline measurements, control variables (gender, age, co-residence with care receiver, income, education, having a life partner, employment and the duration of caregiving) and the intervention as independent variables.

**Results:**

The intervention significantly contributed to the reduction of subjective burden and significantly contributed to the increased likelihood that informal caregivers assumed household tasks. No effects were observed on perceived, health, time investment and quality of life.

**Conclusions:**

This study implies that integrated care models aimed at the frail elderly can benefit informal caregivers and that such interventions can be implemented without demanding additional time investments from informal caregivers. Recommendations for future interventions and research are provided.

**Trial registration:**

Current Controlled Trials http://ISRCTN05748494. Registration date: 14/03/2013.

## Background

Informal caregivers of the frail elderly often experience the demands placed on them as a heavy burden and a threat to their quality of life. Informal care refers to the unprofessional and unpaid assistance provided by partners, family or close friends [1]. Frail elderly people suffer from age-related problems in different domains of daily functioning, such as physical, psychological and social domains, and are at risk of severe problems in the future, such as falls, hospitalization, disability and death [2]. As a result of the myriad of continuously changing problems and the chronic nature of frailty, providing informal care to these patients often entails increasingly intensive care tasks over a prolonged period of time [3]. Referred to as the objective burden of care, such tasks typically require a substantial expenditure of time and energy [4]. Consequently, many informal caregivers experience restrictions on their personal lives as time to spend with friends, to fulfill family obligations or to pursue leisure activities becomes increasingly scarce [5,6]. Informal caregivers may also feel compelled to reduce their working hours, to rearrange their work schedules or to take unpaid leave, affecting their financial situation [5]. Such a multitude of difficulties can lead to an increase in the subjective burden, i.e., the perception of the impact of the objective burden [4]. Moreover, as a result of persistent subjective burden, many informal caregivers perceive deteriorations in their physical health, their social and psychological functioning, their well-being and ultimately their quality of life [7,8].

Despite the potential vulnerability of informal caregivers, their needs are still largely overlooked [9]. Moreover, due to population aging and the trend of replacing institutionally based elderly care with home-based care, informal caregivers are increasingly relied upon [10,11]. Because formal support services for informal caregivers are often inadequate [9], concerns have arisen about the growing burden shouldered by informal caregivers [12]. The involvement of informal caregivers in integrated care arrangements is increasingly considered to benefit both the frail elderly and their informal caregivers [13]. Thus, there has been a trend toward integrated care arrangements that incorporate elderly persons’ entire social systems, including informal caregivers [12]. Integrated care is defined here as a ‘coherent set of methods and models on the funding, and the administrative, organizational, service delivery and clinical levels designed to create connectivity, alignment, and collaboration within and between the cure and care sectors’ [14].

Integrated care arrangements targeting the patient- caregiver dyad are believed to reduce the burden and improve the overall quality of life and health of informal caregivers [12,13,15]. The proactive nature of integrated care is thought to enable the timely recognition of any unmet needs of informal caregivers [16]. Additionally, providing informal caregivers with adequate information (e.g., regarding available services), improving access to care and support services and increasing their competence in coping with their care responsibilities is thought to act as a safeguard against overburdening and deteriorating health [12,17]. Furthermore, it has been argued that certain characteristics of integrated care, such as the emphasis on informal caregiver participation in care planning and provision and increased collaboration with professionals, may result in changes in the division of tasks [18,19]. For instance, informal caregivers are perhaps relieved of some of their more demanding and time-consuming tasks, while enabling them to attend to tasks that are more compatible with their own wishes, their physical abilities and personal lives. Conversely, it has also been suggested that the emphasis on the participation of informal caregivers might actually demand more inputs of time and energy, thereby increasing their burden and ultimately affecting their health and quality of life [1,11,17,20].

However, evidence to substantiate these assumptions is scarce. Whereas the beneficial effects of integrated care on the frail elderly are well established [15], very few studies have reported outcomes for informal caregivers [13]. In a systematic review, Eklund & Wilhelmson [15] found only two studies, both reporting no effect on subjective burden [21,22]. Similarly, Melis et al. [20] reported no effects in terms of both subjective as objective burden. Other authors have described effects of integrated care on informal caregivers, such as reduced caregivers’ stress [23,24], enhanced life satisfaction [25], improved general mental health [26], reduced time investments [17] and, conversely, greater time investments [27,28].

The scarcity and inconsistency of the evidence call for a more coherent and in-depth investigation of the effects of integrated care arrangements on the informal caregiver. To this end, the current study aims to evaluate the effects on informal caregivers of a specific integrated care intervention for the frail elderly, the Walcheren Integrated Care Model (WICM). This model was recently implemented in Walcheren, a region in the southwest of the Netherlands. The current paper describes the investigation of the effects of this intervention on a selection of outcome measures: perceived health, objective burden, subjective burden and quality of life. While it is expected that the WICM will contribute to improvements in these outcome measures, the occurrence of adverse effects as described in existing literature must also be taken into account. Therefore, the research question guiding the current study is formulated accordingly: What are the effects of the WICM on the perceived health, objective burden, subjective burden and quality of life of informal caregivers?

### Intervention

**Figure 1:**
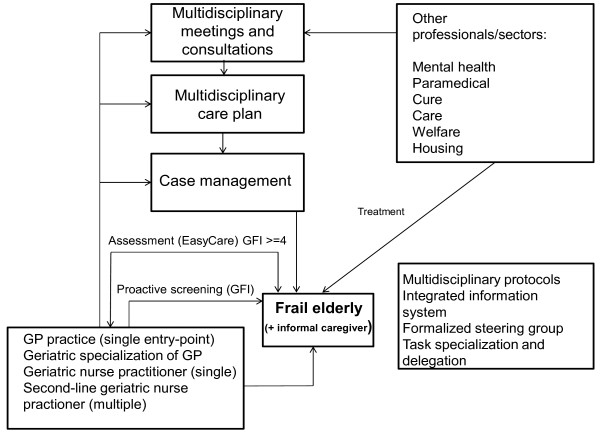
The Walcheren integrated care model.

The WICM focuses on frail elderly individuals living independently (living in their own homes or in a specific type of assisted living facility) and their informal caregivers. The study protocol containing an extensive description of the intervention has been published previously [16]. The WICM has an umbrella organizational structure and includes evidence-based preventive frailty screening and needs assessments of the elderly patient, and needs assessment of the informal caregiver. It contains a single entry point, a multidisciplinary care plan, case management, multidisciplinary consultations and meetings, protocols, a steering group, task specialization/delegation and an integrated information system supporting the entire chain of care (Figure 1).

The WICM entails explicit attention to the potential needs of informal caregivers and recognizes the roles of these individuals in the care process. The involvement of the informal caregiver starts after the patient has been screened for frailty using the Groningen Frailty Indicator (GFI) [29]. After being identified as frail, patients are visited by a case manager who performs a comprehensive assessment of needs using an evidence-based instrument. In this phase, the informal caregiver’s needs for support and guidance are also identified. The case manager determines the care goals in consultation with the care recipient and the informal caregiver, after which a care plan is formulated. Consequently, the plan is discussed, refined and approved in a multidisciplinary meeting. The general practitioner (GP) contacts the care recipient and informal caregiver to provide the opportunity for any last adjustments. A case manager implements the care plan and coordinates care delivery. Periodic evaluations of the care plan ensure adequate monitoring of the needs of the care recipient and the informal caregiver.

Available services for informal caregivers normally include respite care services aimed at temporary relief, as well as psychosocial interventions, such as education and training or (group) counseling. In the WICM, the case manager provides the informal caregiver with relevant information, advice and suggestions regarding available services based on the caregiver’s specific needs. The case manager functions as a link to all relevant organizations and professionals and if needed, the informal caregiver is brought into contact with them. Case managers may also provide practical advice (e.g., how to make certain care tasks less burdensome) or emotional support.

## Methods

### Study design and participants

The study had a quasi-experimental design included before/after measurements and a control group. A baseline measurement (T0) was performed prior to the intervention; the follow-up measurement (T1) was performed twelve months after T0. The study protocol (protocol number MEC-2013-058) was reviewed by the medical ethics committee of the Erasmus Medical Centre Rotterdam, the Netherlands. They waived further examination as the Medical Research Involving Subjects Act did not apply.

Eight GP practices in the Walcheren region participated in this study as intervention practice or control practice. Frail older patients and their informal caregivers were recruited as participants through these practices. Both control and experimental practices provided the researchers with the names and contact information of patients that were 75+ years of age. These patients were mailed an information leaflet, the screening questionnaire (GFI) and an informed consent. Upon return, frailty scores were computed (GFI score of 4+) [29]. Inclusion followed if patients did not meet the exclusion criteria of being terminally ill and living in a nursing home. Subsequently, their informal caregivers were recruited by asking the included frail older patients whether they received informal care and, if so, from whom. It was explained to patients that informal care involves all non-professional and unpaid assistance provided by partners, family or close friends and neighbors. The informal caregivers were then mailed an information leaflet and informed consent, which they were asked to fill out and return.

Of the 8 participating GP practices, 3 practices (6 GPs) provided care according to the WICM and constituted the experimental group. The remaining 5 practices (5 GPs) continued to provide care as usual and thus constituted the control group. Care as usual for the frail elderly can be described as reactive, as GPs are usually consulted at the patient’s initiative. As gatekeepers, GPs refer frail elderly patients to both care and curative services in the secondary and tertiary echelons [30]. Care as usual does not include case management or formal multidisciplinary collaboration.

### Data collection

The questionnaire [see Additional file 1] was developed as part of a large-scale national program initiated by the Ministry of Health, Welfare and Sports [31]. With a budget of 80 million euros, This National Care for the Elderly Program (NPO) aims to improve care for the elderly by initiating interventions and providing platforms for the dissemination of study results. All interventions operating within the NPO-program use the questionnaire, thereby ensuring optimal data-sharing [32]. Data were collected by trained interviewers who visited participating patients at home. If the informal caregiver was present, the data were collected in a face-to-face interview. If not, questionnaires were sent by mail to the informal caregiver’s home address. All interviewers had previously worked in elderly care and lived in the region.

### Outcome measures

No hierarchical division of outcome measures into primary and secondary outcomes was made in the current study. Perceived health was measured using 2 items from the RAND-36 [33]. On the first item, the respondent indicates his or her current perceived health on a 5-point Likert scale ranging from 1 (poor) to 5 (excellent). On the second item, the respondent indicates the changes in perceived health in comparison to 12 months ago on a 5-point Likert scale ranging from 1 (a lot worse) to 5 (a lot better).

Objective burden was measured with the short form of the ‘Objective Burden of Informal Care Instrument’ [34]. This instrument operationalizes objective burden as the amount of time spent and the nature of care tasks. Thus, respondents indicate the nature of performed tasks (household, personal care and instrumental care tasks) and the amount of time spent on each category of tasks during the week of measurement. In addition, respondents indicate whether other informal caregivers provide assistance and if so, what their time investments are.

Subjective burden was measured with the CarerQoL [35], the Process Utility (PU) Scale [36] and the Self-Rated Burden (SRB) Scale [37]. While all 3 instruments aim to measure subjective burden, their approaches differ and thus these instruments are considered to be complementary to each other. The CarerQoL describes the caregiver’s situation in terms of both positive and negative aspects of informal care, thereby providing a balanced measure of subjective burden. Negative aspects are the experience of problems in physical health, mental health, financial situation, relationships and in combining care tasks with personal activities. Positive aspects are the experience of support from others and feelings of fulfillment. Respondents indicate the degree to which each aspect is applicable to their current situation (response categories: none/some/a lot). A weighted sum score (0–100) describes the specific caregiver’s situation, in which a higher sum score indicates a more favorable situation. In addition, the CarerQoL includes a visual analog scale (VAS) that provides an indication of the current general happiness ranging from 0 (completely unhappy) to 10 (completely happy). The VAS for process utility (PU) provides a measure for the respondent’s happiness derived from caregiving. Respondents indicate their degree of happiness ranging from 0 (completely unhappy) to 10 (completely happy) with a hypothetical scenario in which all care tasks are assumed by a professional caregiver. The final measure for subjective burden is the SRB, a VAS ranging from 0 (not at all burdensome) to 10 (way too burdensome), indicating the degree to which informal care is experienced as burdening.

Quality of life was measured using Cantril’s Self-Anchoring Ladder [38]. The respondents rate their current quality of life on a scale from 0 to 10. Two additional items were used to assess quality of life and changes in quality of life in comparison with 12 months ago. These items were based on the items on perceived health from the RAND-36 [33]. Just as the items for perceived health, respondents indicate their current quality of life on a 5-point Likert scale ranging from 1 (poor) to 5 (excellent) and the changes in quality of life in comparison to 12 months ago on a 5-point Likert scale ranging from 1 (a lot worse) to 5 (a lot better).

### Control variables

Literature indicates that being female, being older, having a lower level of education, having a low income, the relationship to the care-recipient (child versus spouse), co-residence with the care recipient, being employed and providing informal care for a longer duration of time increases the informal caregiver’s burden [1,39-41]. Thus these factors served as control variables in the current study. The level of education was assessed using Verhage’s categorization [42]. Income was assessed relative to the average income in the Netherlands in 2010 (33,500 €) on a five-point scale from 1 (much less than 33,500 €) to 3 (approximately 33,500 €) to 5 (much more than 33,500 €).

### Analysis

#### Transformations

A number of outcome measures required transformations prior to analysis. The items for perceived health (RAND-36) and the items for quality of life (based on the RAND-36 items) were reversely recoded so that a higher score signified better health and quality of life. As specified in the RAND-36 manual [33], the 5-point Likert scale was converted into a 100-point scale. As for the CarerQoL [35], the negative dimensions were assigned the values 0 (a lot), 1 (some) and 2 (none); the positive dimensions were assigned the values 0 (none), 1 (some) and 2 (a lot), so that high scores signified higher well-being. Process utility was derived through the computation of a difference score between the CarerQoL-VAS (happiness now) and the PU-VAS (happiness if care tasks are taken over a by professional) resulting in a score ranging from −10 to 10. In addition, to enable the inclusion of the control variables income and education in further analyses, these variables were transformed into dichotomous variables (with values ‘low’ and ‘high’) by creating groups of approximately equal size.

#### Within- and between group analyses

Mean scores were computed for all outcome measures and were subsequently analyzed using t-tests, thus providing a description of the scores of the groups at T0 and T1. Specifically, within-group changes between T0 and T1 were determined using a paired t-test, McNemar’s test or Wilcoxon’s signed rank test. To compare scores between groups, difference scores were computed for all outcome measures, which were then analyzed using independent t-tests and chi-square tests (or Fisher’s exact test). Significant effects indicate that changes in scores between T0 and T1 differ substantially between groups.

#### Regression analyses

To further investigate the contribution of the intervention to the observed differences in scores between groups, regression analyses were performed. Linear regression analyses were used for the outcome variables perceived health, subjective burden, quality of life and amount of time spent, while logistic regression analyses were performed on the binary variables related to objective burden i.e., informal caregivers performing household tasks, personal care tasks and instrumental care tasks. Regression analyses consisted of 3 consecutive models containing the baseline scores of the specific outcome variable (Model 1), control variables age, gender, income level, education level, co-residence, employment, having a life partner and the duration of caregiving in months (Model 2) and the intervention (Model 3). As the regression analyses aimed to assess the contribution of the intervention, controlling for baseline scores and control variables, only the output of Model 3 (coefficients and significance) is reported in this paper.

Models and effects of the WICM were considered significant if *p* < 0.05. However, as the definitive study sample was relatively small, p-values of < 0.1 were also reported [43]. Additionally, to determine the degree of multicollinearity between control variables, the values of tolerance (< 0.2) and the variance inflation factor (> 10) were checked [44]. This revealed that multicollinearity indeed existed between the variables ‘relationship to care recipient’ (child versus spouse) and ‘co-residence with the care recipient’. Consequently, it was decided to drop the variable that explained the least amount of variance i.e., the variable ‘relationship to care recipient’.

## Results

At T0, a total of 377 patients were included as a participant in the WICM (Table 1). The majority of patients was female, had an average age of 82 years and an average frailty score (GFI) of around 6. Most patients did not have a partner (anymore) and most lived independently. Comparison of the care recipient characteristic between groups revealed that the percentage of female care recipients was significantly higher in the experimental group than in the control group. In addition, the experimental group consisted of significantly more care recipients with assisted living arrangements or that lived in a nursing home.

**Table 1:** **Characteristics of care recipients and caregivers and loss to follow**-**up**

**Characteristics of care recipients**			
**Background variables**	**Experimental group (N=184)**	**Control group (N=193)**	**Total (N=377)**
Frailty (GFI score)	6.0 (2.0)	5.8 (1.8)	5.9
Female*	70%	60%	65%
Age	81.8 (SD: 4.7)	82.3 (SD: 5.3)	82
Partner (married or co-residing)	37%	42%	39%
Single (or widowed)	63%	58%	61%
Independent living	72%	82%	77%
Assisted living/nursing home*	28%	18%	23%
Receiving informal care	144 (78.3%)	118 (61.1%)	262 (69.5%)
*Caregiver loss to follow*-*up*	61	42	103 (39.3%)
*Caregivers participating*	83	76	159
**Characteristics of informal caregivers**			
**Background variables**	**Experimental group (N=83)**	**Control group (N=76)**	**Total (N=159)**
Female	71.0%	75.0%	73%
Age*	60.7 (SD: 12.2)	65.6 (SD: 11.2)	63.2
Co-residing with care recipient	28.9%	40.8%	34.9%
Relationship to care recipient:			
*Partner*	26.5%	36.8%	31.6%
*Son/daughter*	68.7%	51.3%	60.0%
*Other (e.g. neighbor, friend)*	4.8%	11.9%	8.4%
Low education	65.4%	66.2%	65.8%
High education	34.6%	33.8%	34.2%
Low income	58.0%	65.8%	61.9%
High income	42.0%	34.2%	76.2%
Having life partner	89.0%	88.2%	88.6%
Employed (yes)	50.0%	38.4%	44.2%
Duration (in months)	92.8 (SD: 93.8)	97.3 (SD: 115.7)	95.1

*p < 0.05.

Of the total of 377 care recipients, 262 indicated to receive care from an informal caregiver. However, due to a loss to follow-up (N = 103), this number had reduced to a total of 159 at T1. The majority of these losses to follow-up were due to informal caregivers not responding after the initial contact (N = 53/103). Others were unwilling to continue to participate in the study (N = 16/103) or felt the definition of informal caregivers did not apply to them (N = 15/103). Some of these informal caregivers indicated that their care tasks had been taken over by formal caregivers since the baseline measurement, while others considered their caregiver role as their duty rather than deserving of a distinctive label. Finally, a number of losses to follow-up were the result of the progressive inability or death of the care recipient (N = 19/103). The definitive study population of informal caregivers consisted of all respondents of which data were available for both T0 and T1. This amounted to 83 informal caregivers in the experimental group and 76 informal caregivers in the control group.

Subsequent comparison between groups on control variables and baseline scores on all variables showed that informal caregivers in both groups were equal except on the variable age. Specifically, the mean age of informal caregivers in the control group was significantly higher than the mean age of informal caregivers in the experimental group. In general, the age of informal caregivers in the study population was 63 years. A large majority was female, and most had a life partner. In addition, most had a low educational level and a low income. Sons and daughters (in law) constituted the largest group of informal caregivers, followed by partners. Half of the informal caregivers in the experimental group and nearly 40% in the control group were employed during the study period. The average duration of caregiving in both groups was approximately 8 years. Around one-third of informal caregivers in both groups co-resided with the care recipient.

### Within-group and between-groups differences

#### Perceived health

While both the experimental and the control group showed a decline in perceived health between T0 and T1, only the decline in the control group was significant (*p* = 0.007). Subsequent analysis of difference scores showed a moderately significant difference between groups (*p* = 0.087) (Table 2).

**Table 2:** **Within**-**group and between-group differences in mean scores at T0 and T1**

**Outcome variables**	**Experimental group**		**Control group**		**Between-groups comparison**
	**T1**	**Δ T0**	**T1**	**Δ T0**	Δ
**Perceived health**					
Perceived health (0–100)	46.91	−1.23	44.00	−6.33*	#
Perceived change in health (0–100)	46.30	−2.16	46.00	−2.00	-
**Subjective burden**					
CarerQoL sum score (0–100)	84.93#	3.88*	80.73	−0.55	*
CarerQoL-VAS (0–10)	7.16	−0.07	6.97	−0.49*	#
Process Utility (−10-10)	2.59	−0.09	2.38	−0.71#	-
Self -Rated Burden (SRB) Scale (0–10)	3.97	0.54#	3.95	0.63#	-
**Objective burden**					
% of caregivers performing household tasks	87.2%	7.7%	76.7%	−1.4%	-
% of caregivers performing personal care tasks	30.5%	4.9%	41.3%	14.6%*	-
% of caregivers performing instrumental care tasks	79.3%	−4.8%	69.7%	−6.6%	-
% reporting other informal caregivers	45.0%	6.2%	34.2%	4.1%	-
Hours spent per week on household tasks	7.25	1.46	8.93	2.44#	-
Hours spent per week on personal care tasks	1.86	0.76#	2.17	0.50	-
Hours spent per week on instrumental care tasks	2.51	0.46	1.79	−0.43	-
Total hours spent per week	11.15	2.44#	12.53	2.25	-
Total hours spent per week (incl. other caregivers)	13.25	3.14*	13.03	1.57	-
**Quality of life**					
Quality of life (0–100)	55.63	−1.87	54.67	−5.67*	-
Change in quality of life (0–100)	48.15	−4.63#	46.33	−4.67#	-
Rating of quality of life (0–10)	7.35	−0.04	7.37	−0.29*	-

#*p* < 0.10; *p < 0.05; **p < 0.01; ***p = 0.000; “-“= no significance; Δ T0 = difference between T1 and T0; Δ = difference between control and experimental groups.

#### Subjective burden

Measures used to assess the effects of the intervention on subjective burden were the CarerQoL sum score and VAS, Process Utility (PU) and the Self-Rated Burden Scale (SRB). Although these measures yielded somewhat mixed scores, overall, results were more favorable for the experimental group. The experimental group showed a significant improvement of CarerQoL sum scores between T0 and T1 (*p* = 0.008), while the control group showed a slight (non-significant) reduction of CarerQoL sum scores. Both groups showed reductions in CarerQoL-VAS scores between T0 and T1, although the reduction was only significant for the control group (*p* = 0.008). PU scores did not change between T0 and T1 for the experimental group, while the control group showed a moderately significant reduction of PU scores (*p* = 0.071). Both the experimental group (*p* = 0.057) as the control group (*p* = 0.072) showed moderately significant increases in SRB score between T0 and T1. Comparison of the within-group differences over time revealed a significant difference between groups for the CarerQoL sum score (*p* = 0.033) and a moderately significant difference between groups for the CarerQoL-VAS (*p* = 0.060).

#### Objective burden

Objective burden constituted the number of hours that informal caregivers spent on care and the categories of care tasks. The number of hours spent on household tasks increased in both groups between T0 and T1. However, only the increase in the control group was moderately significant (*p* = 0.084). Similarly, both groups showed an increase in the number of hours spent on personal care tasks, although the increase was only moderately significant for the experimental group (*p* = 0.094). Both groups showed no significant changes in the hours spent on instrumental care tasks. The total time investment also increased significantly for the experimental group, both including additional informal caregivers (*p* = 0.045) and excluding additional informal caregivers (*p* = 0.067). The control group showed a significant and substantial increase between T0 and T1 in the percentage of caregivers performing personal care tasks (*p* = 0.013). However, none of these changes over time within the groups resulted in significant differences between groups.

#### Quality of life

While the control group showed decreased scores on all 3 items for quality of life between T0 and T1, the experimental group showed a decreased score on 1 item only. Specifically, the control group showed reductions in perceived quality of life (*p* = 0.023), adverse changes in quality of life (*p* = 0.080) and in overall ratings of quality of life (*p* = 0.032). The experimental group only showed a moderately significant adverse change in quality of life (*p* = 0.071). No significant differences between groups were observed on these items.

### Regression analysis

The WICM resulted in a significant reduction of subjective burden (*p* = 0.053) as measured with the CarerQoL sum score. In addition, logistic regression analyses showed that the WICM significantly increased the likelihood of informal caregiver’s performing household tasks (*p* = 0.048). The intervention showed no effects on the outcomes perceived health and general quality of life (Table 3).

**Table 3:** **Regression**/**logistic regression analyses with baseline scores, Control Variables and the Intervention as Predictors**

**Outcome Variables**	**Independent variables**									
	**T0**	**Gender**	**Age**	**Co-residence**	**Employment**	**Partner**	**Education**	**Income**	**Duration**	**WICM**
**Perceived health**										
Perceived health (0–100)	0.673***	−**0.134**#	−0.73	−**0.190**#	−0.102	0.059	−0.112	0.061	−0.036	0.079
Perceived change in health (0–100)	**0.148**#	−0.226*	−0.092	−0.396**	−**0.181**#	0.008	−0.005	0.084	−0.216**	−0.043
**Subjective burden**										
CarerQoL sum score (0–100)	0.571***	−0.219*	0.144	−0.261*	−0.053	−0.090	−**0.135**#	−0.003	−0.042	**0.132**#
CarerQoL-VAS (0–10)	0.562***	0.013	0.108	−**0.216**#	0.034	0.052	0.012	−0.066	0.045	0.096
Process Utility (PU) (−10-10)	0.605***	−0.067	−0.034	−0.135	−0.069	0.018	−0.031	−0.076	0.222*	0.041
Self-Rated Burden (SRB) Scale (0–10)	0.373***	0.288**	0.151	0.069	0.118	0.119	0.021	0.059	0.017	0.052
**Objective burden**										
Caregivers performing household tasks (log)	8.795***	3.345	1.088	0.157	1.311	0.129	1.313	0.200*	1.000	3.590*
Caregivers performing personal care tasks (log)	10.357***	2.458	1.023	5.829*	1.493	1.045	2.164	0.654	1.000	0.666
Caregivers performing instrumental care tasks (log)	12.825***	4.785*	0.964	1.433	0.444	2.560	0.782	2.133	1.003	1.281
Additional informal caregivers (log)	9.929***	1.040	1.171	0.157*	1.608	0.379	**2.314**#	0.980	0.998	1.171
Hours spent on household tasks	0.457***	0.009	0.064	0.278*	−0.026	−0.166*	0.058	−0.060	−0.192**	0.020
Hours spent on personal care tasks	0.569***	0.007	0.049	0.188	−0.006	0.050	0.007	0.004	−0.022	0.009
Hours spent on instrumental care tasks	-	-	-	-	-	-	-	-	-	-
Total hours spent	0.577***	0.032	0.132	0.146	−0.003	−0.101	0.033	−0.024	−0.137*	0.071
Total hours spent + other caregivers	0.585***	0.041	0.108	0.166	0.042	−0.084	0.087	−0.042	−0.118	0.107
**Quality of life**										
Quality of life (0–100)	0.361***	−**0.161**#	−0.041	−0.421**	−0.096	0.106	−0.034	0.056	−0.013	−0.046
Change in quality of life (0–100)	-	-	-	-	-	-	-	-	-	-
Rating of quality of life (0–100)	0.318***	0.097	0.136	−0.320*	−0.010	0.037	−0.007	**0.155**#	−0.001	0.059

#*p* < 0.10; *p < 0.05; **p < 0.01; ***p = 0.000 (significant coefficients/Exp(B) shown in bold ); (log) = logistic regression analyses; WICM = Walcheren Integrated Care Model.

Note: Positive Beta values signify better scores for all outcomes with a 0–10 or 0–100 range, except for the outcome Self-Rated Burden (SRB); for SRB, positive Beta values signify increased experienced burden.

Baseline scores were the dominant predictors for all outcomes, followed by co-residence and gender. Co-residence negatively affected perceived health, general quality of life, subjective burden (CarerQoL sum score and VAS) while it increased the amount of hours spent on household tasks. Additionally, co-residence resulted in increased likelihood of informal caregivers performing personal care tasks. Female caregivers experienced higher subjective burden (as measured by CarerQoL sum scores and SRB scores) and were more likely to perform instrumental care tasks than male caregivers. Additionally, female caregivers perceived their health and general quality of life to be poorer than male caregivers. The longer informal caregivers provided care, the poorer they perceived their health to be. Conversely, longer periods of caregiving reduced the amount of hours spent on household tasks and increased the process utility of caregiving. A higher income enhanced the general quality of life ratings and increased the likelihood of caregivers performing household tasks. A higher education increased the amount of hours spent by other informal caregivers and increased the subjective burden (CarerQoL sum score). Having a life partner reduced the number of hours spent on household tasks and the number of hours spent by other informal caregivers. Being employed resulted in more hours spent by other informal caregivers and poorer perceived health. The regression models for instrumental care tasks and changes in quality of life were not significant. Additional file 2 summarizes the significance and the contribution to the explained variance of each regression model. This overview confirms that Model 1 (baseline scores) explained the greatest proportion of the variance, followed by Model 2 (control variables). Model 3 (the intervention) contributed relatively little to the explained variance.

## Discussion

This study explored the effect of the WICM on the perceived health, objective burden, subjective burden and the general quality of life of informal caregivers of frail elderly patients. Our results show that the WICM reduced the subjective burden of informal caregivers. In addition, the likelihood of informal caregivers assisting with household tasks increased as a result of the WICM.

The reduction of subjective burden that was observed in this study was measured with the CarerQoL, an instrument that describes the caregiver’s situation in 7 common dimensions of informal care provision. Thus, this positive outcome indicates that informal caregivers experienced fewer problems and more support and satisfaction as a result of the intervention. Certain components of the WICM may have contributed to this positive outcome, such as the proactive character, the needs assessment, explicit attention to the support of informal caregivers and periodic monitoring. Etters et al. [45] concluded that prevention, early detection and periodic screening are effective in identifying informal caregivers at risk of being overburdened. Similarly, Sörensen et al. [46] emphasized the importance of targeting unmet needs and providing opportunities for respite care. In addition, others have emphasized that the dynamic needs of the informal caregivers of the frail elderly require regular monitoring [47,48]. Although the current study suggests that these components have contributed to the improvements in the informal caregiver’s situation, they may have asserted their effect independently or interdependently and thus causality cannot be determined [49]. Alternative explanations may be provided by the possibility of improved patient outcomes and improved relationships between the patient and the informal caregiver. An abundance of evidence exists of the beneficial effects of integrated care on the elderly patient’s physical abilities, functional abilities and well-being [50]. There is also evidence that such improvements can result in less intensive and exhausting informal care tasks, thereby reducing the informal caregiver’s distress [51,52]. In the context of improved relationships, Schultz and Martire [40] noted that informal caregiving occurs by definition in a social context and that informal caregiver outcomes cannot be viewed separately from the relationship with the care recipient. A reciprocal negative affect between spousal care recipients and informal caregivers has been previously described [53,54], suggesting an association between the quality of the relationship and outcomes for caregivers.

A second finding of this study was that although the WICM did not affect the time investments of informal caregivers, it did increase the likelihood of informal caregivers assuming household tasks. Although changes in tasks were considered as possible outcome of the WICM, the direction of such changes was unclear. As described in the background section of this paper, this may have been the outcome of changes in the division of tasks between professionals and informal caregivers [18,19]. The emphasis on the informal caregiver’s participation in care planning and provision might have resulted in a ‘negotiation process’ between the case manager and the caregiver, through which the latter may have become more aware of his or her role in the care process. Perhaps this has prompted informal caregivers to take up those tasks that can easily be performed by non-professionals, such as household tasks. Alternatively, it has been suggested that the care recipient’s health status affects the nature of informal care tasks [55]. It can thus be argued that the shift towards household tasks observed in the current study may have been the result of changes in the degree of impairment of the care recipient.

The finding that the time investments were not affected by the WICM is consistent with the findings of previous research [11,20], although other studies suggest that integrated care increases time investments of informal caregivers [17,27]. Weuve et al. [11] provided an explanation, suggesting that certain intervention components (e.g. case management, training or consultation) may increase the competence of informal caregivers, thereby buffering the potential increase in time investment.

No effect was found on the perceived health of the informal caregivers. This observation might be explained by the relative stability of self-rated health over time [56]. A study period of 12 months may be too short to observe meaningful changes in perceived health. Similarly, no effects were found on the general quality of life. This result is somewhat unexpected as previous studies have demonstrated the existence of an association between subjective burden and quality of life of informal caregivers [7,8,39]. The failure of the current study to observe such an association may have been the result of the use of non-validated measures for quality of life.

Overall, it can be argued that the effects of the WICM on informal caregivers are promising but modest. Several factors may have somehow inhibited the effectiveness of the intervention. First, the majority of informal caregivers did not co-reside with the care recipients. Perhaps these informal caregivers were less affected by the intervention, which would mitigate its effectiveness. It has been argued that integrated care interventions aimed at the frail elderly may be less appropriate for certain subgroups of informal caregivers, such as those who do not live with the care recipient [48]. If so, integrated care interventions that allow a more flexible approach to informal caregivers could be more effective, for instance by applying different strategies for different subgroups. Second, it is possible that the modest results of the current study are related to a suboptimal implementation of the intervention which could have resulted in a limited exposure of the target populations to the intervention [57]. It can be argued that the evaluation period of 12 months used in this study may have been too short for the intervention to reach its full potential. If so, stronger effects can be expected if longer evaluation periods are used as the likelihood of interventions affecting informal caregivers increases [58]. Some rationale for such long-term effects is provided by the results of the within- and between-group analyses. The observed deteriorations in perceived health and happiness (CarerQoL-VAS) were larger in the control group than in the experimental group, which resulted in significant between-group differences. While the regression analyses showed that the intervention did not contribute to these group differences over a 12-month period, perhaps the contribution of the intervention might increase over a longer period of time. Specifically, it provides some basis for the hypothesis that integrated care interventions such as the WICM may protect informal caregivers against the natural decline in health and well-being that is associated with providing care in the context of the progressive trajectory of frailty. However, identifying such long-term effects requires a control group that remains intact over an extended period. This might prove to be difficult as over time the control group might become ‘contaminated’ when certain elements of integrated care are adopted into the control condition.

### Limitations

The use of non-validated items for general quality of life is a limitation of this study. However, the questionnaire was developed by an expert group, which considerably enhanced its face validity. The relatively low proportion of variance that was explained by the intervention constitutes another limitation. This is especially relevant in light of the moderate significance of the effect that was observed in this study and thus, this result must be interpreted with caution. Another limitation is the relatively low contribution of the control variables to the explained variance, suggesting that additional variables need to be taken into account. For instance, the degree of frailty and the nature of disability of the care recipients might be of influence [40]. All patients in the current study were identified as frail based on their frailty scores and were thus considered to be fairly similar in terms of their disabilities. However, as frailty scores showed some variation in the patient group, they have asserted influence on the outcomes. While the current study only used caregiver characteristics as control variables, including frailty scores as a control variable might have yielded more robust regression models. Additionally, dysfunctional family relations, personality traits of the informal caregiver or preexisting medical conditions have been proposed as mediating factors [46]. Another limitation is the relatively large loss to follow-up, which increases the risk of selection bias and threatens the generalizability of the study results. These substantial losses justify a post-hoc analysis of the non-response group to determine their characteristics. However, as around 50% of losses occurred prior to baseline measurement, the data needed to assess the effect of the loss to follow-up were not available. Nonetheless, it is conceivable that the individuals that dropped out of the study were actually the most burdened and in greatest need of a supportive intervention [48]. A final limitation of this study is the large number of statistical tests that were performed without applying corrections for multiple comparisons.

### Recommendations

Future research is recommended to focus on matching intervention components to informal caregiver outcomes. Research should also focus on the associations between improvements in the abilities of the elderly, the quality of the relationship and the outcomes for informal caregivers in integrated care interventions. Future research is recommended to investigate which aspects of integrated care interventions lead to specific shifts in tasks, especially the shift toward household tasks as observed in this study. Furthermore, the effect of integrated care on the time investment of informal caregivers requires further investigation. Future studies should consider using evaluation periods longer than 12 months to increase the likelihood of observing more robust effects. A longer time-frame would provide more opportunity to allow a start-up period for an optimal implementation of the intervention, in which all actors can become accustomed to new working arrangements. Finally, integrated care interventions aiming at both the frail elderly and their informal caregivers may be more effective when differentiation in the approach of subgroups can be made, particularly in regard to co-residing and non-co-residing informal caregivers.

### Study strengths

Very few studies have specifically aimed to evaluate the effects of an integrated care intervention on informal caregivers. This study aimed to fill this gap by using a sound study design, a broad range of control variables, outcome variables and several validated instruments.

## Conclusions

Our main conclusion is that the WICM reduced the subjective burden by improving the situation of the informal caregivers and increased the likelihood of informal caregivers assisting with household tasks. Our results indicate that integrated care interventions can benefit informal caregivers. In addition, this study shows that time investments of informal caregivers do not necessarily increase as a result of integrated care. We believe that this finding should be interpreted as a positive outcome. Integrated care has been shown to benefit the frail elderly, to improve the quality of care and to reduce costs. This study indicates that these outcomes can be achieved while reducing the subjective burden and retaining the level of commitment of informal caregivers. Given the increasing pressure on informal caregivers of the frail elderly, it is vital to find effective means to support these individuals. Our findings indicate that integrated care can be a viable approach to do so.

## Abbreviations

WICM: Walcheren Integrated Care Model; GP: General Practitioner; SRB: Self-Rated Burden; VAS: Visual Analogue Scale; PU: Process Utility; CarerQoL: Care-Related Quality of Life; NPO: Nationaal Programma Ouderenzorg (National Care for the Elderly Programme); GFI: Groningen Frailty Indicator.

## Competing interests

The authors declare that they have no competing interests.

## Authors’ contributions

RK was one of the main initiators of the Walcheren Integrated Care Model and was responsible for its design and ensuring commitment of all parties involved. BJ collected the data, performed the statistical analyses and wrote the paper. As the project leader of the study, IF planned and designed the study, supervised the data collection and analyses and contributed to revising the paper. RH contributed to revising the paper. All of the authors read and approved the final manuscript.

## Pre-publication history

The pre-publication history for this paper can be accessed here:

http://www.biomedcentral.com/1471-2318/14/58/prepub

## Supplementary Material

Additional file 1 Description of file: English version of the questionnaire used in the current study (developed for the ‘Nationaal Programma Ouderenzorg’ (NPO) [National Care for the Elderly Program]. Click here for file

Additional file 2 Description of file: Table showing the contribution of each model to the R2 and their significance. Click here for file

## References

[B1] Carretero S Garcés J Ródenas F Sanjosé V The informal caregiver's burden of dependent people: Theory and empirical review Arch Gerontol Geriatr 2009 14 1 74 79 18597866 10.1016/j.archger.2008.05.004

[B2] Gobbens RJ Luijkx KG Wijnen-Sponselee MTH Schols JMGA Towards a conceptual definition of frail community-dwelling older people Nurs Outlook 2010 14 2 76 86 20362776 10.1016/j.outlook.2009.09.005

[B3] Smale B Epp T Dupuis SL Caregivers of persons with dementia: Roles, experiences, supports and coping: A literature review 2004 Ontario: Murray Alzheimer Research and Education Program: University of Waterloo

[B4] Brouwer WBF Van Exel NJA van den Berg B Dinant HJ Koopmanschap MA van den Bos GAM The burden of caregiving: Evidence on objective burden, subjective burden and quality of life impacts in informal caregivers for patients with Rheumatoid Arthritis Arthritis Care Res 2004 14 4 570 577 10.1002/art.20528 15334429

[B5] Stone RI Farley P The competing demands of employment and informal caregiving to disabled elders Med Care 1990 14 6 513 526 2355757 10.1097/00005650-199006000-00004

[B6] Gupta R Pillai VK Elder caregiving in South-Asian families in the United States and India Soc Work Soc 2012 14 2

[B7] Ekwall A Sivberg B Hallberg IR Dimensions of informal care and quality of life among elderly family caregivers Scand J Caring Sci 2004 14 3 239 248 15355517 10.1111/j.1471-6712.2004.00283.x

[B8] Coen RF O'Boyle CA Coakley D Lawlor BA Individual quality of life factors distinguishing low-burden and high-burden caregivers of dementia patients Dement Geriatr Cogn Disord 2002 14 3 164 70 11893838 10.1159/000048648

[B9] Lopez-Hartmann M Wens J Verhoeven V Remmen R The effect of caregiver support interventions for caregivers of community-dwelling frail elderly: A systematic review Int J Integr Care 2012 14 5 e133 23593047 10.5334/ijic.845 PMC3601532

[B10] Navaie-Waliser M Feldman PH Gould DA Levine C Kuerbis AN Donelan K When the caregiver needs care: The plight of vulnerable caregivers Am J Public Health 2002 14 3 409 413 11867321 10.2105/ajph.92.3.409 PMC1447090

[B11] Weuve JL Boult C Morishita L The effects of outpatient geriatric evaluation and management on caregiver burden Gerontologist 2000 14 429 36 10961032 10.1093/geront/40.4.429

[B12] McAdam M Frameworks of integrated care for the elderly: A systematic review 2008 Ontario: Research Report, Canadian Policy Research Networks

[B13] Hallberg IR Kristensson J Preventive home care of frail older people: A review of recent case management studies J Clin Nurs 2004 14 112 20 15724826 10.1111/j.1365-2702.2004.01054.x

[B14] Kodner D Spreeuwenberg C Integrated care: Meaning, logic, applications and implications – A discussion paper Int Integr Care 2003 14 3 10.5334/ijic.67 PMC1480401 16896389

[B15] Eklund K Wilhelmson K Outcomes of coordinated and integrated interventions targeting frail elderly people: A systematic review of randomized controlled trials Health Soc Care Community 2009 14 447 458 19245421 10.1111/j.1365-2524.2009.00844.x

[B16] Fabbricotti IN Janse B Looman WM Kuijper De R Wijngaarden Van JDH Reiffers A Integrated care for frail elderly compared to usual care: A study protocol of a quasi-experiment on the effects on the frail elderly, their caregivers, health professionals and health care costs BMC Geriatrics 2013 14 31 10.1186/1471-2318-13-31 PMC3648376 23586895

[B17] Wimo A Von Strauss E Nordberg G Sassi F Johansson L Time spent on informal and formal caregiving for persons with dementia in Sweden Health Policy 2002 14 255 268 12098519 10.1016/s0168-8510(02)00010-6

[B18] Paulus ATG Van Raak A Keijzer F Informal and formal caregivers’ involvement in nursing home care activities: Impact of integrated care J Adv Nurs 2005 14 354 366 15701150 10.1111/j.1365-2648.2004.03299.x

[B19] Noelker LS Bass DM Home care for elderly persons: Linkages between formal and informal caregivers J Gerontology: Soc Sci 1989 14 2 S63 70 10.1093/geronj/44.2.s63 2921480

[B20] Melis RJF Van Eijken MIJ Van Achterberg T Teerenstra S Vernooij-Dassen MJFJ van de Lisdonk EH The effect on caregiver burden of a problem-based home visiting programme for frail older people Age Ageing 2009 14 5 542 547 19574322 10.1093/ageing/afp101

[B21] Montgomery P Fallis W South Winnipeg Integrated Geriatric Program (SWING): a rapid community response program for the frail elderly Canadian Journal of Aging 2003 14 3 275 281

[B22] Beland F Bergman H Lebel P Clarfield AM Tousignant P Contandriopoulos A Dallaire L A system of integrated care for older persons with disabilities in Canada: results from a randomized controlled trial The Journals of Gerontology Series A – Biology Sciences and Medical Sciences 2006 14 4 367 373 10.1093/gerona/61.4.367 16611703

[B23] Tibaldi V Aimonino N Ponzetto M Amati D Raspo S Roglia D Molaschi M Fabris F A randomized controlled trial of a home hospital intervention for frail elderly demented patients: Behavioral disturbances and caregiver’s stress Arch Gerontol Geriatr 2004 14 Suppl 431 436 10.1016/j.archger.2004.04.055 15207444

[B24] Silverman M Musa D Martin DC Lave JR Adams J Ricci EM Evaluation of outpatient geriatric assessment: A randomized multi-site trial J Am Geriatr Soc 1995 14 7 733 740 7602022 10.1111/j.1532-5415.1995.tb07041.x

[B25] Hung LC Liu CC Hung HC Kuo HW Effects of a nursing intervention program on disabled patients and their caregivers Arch Gerontol Geriatr 2003 14 3 259 272 12849081 10.1016/s0167-4943(02)00170-x

[B26] Smits CH De Lange J Droes RM Meiland F Vernooij-Dassen M Pot AM Effects of combined intervention programmes for people with dementia living at home and their caregivers: A systematic review Int J Geriatr Psychiatry 2007 14 1181 1193 17457793 10.1002/gps.1805

[B27] Braun K Rose C Testing the impact of a case management program on caregiver appraisal J Gerontol Soc Work 1994 14 3/4 51 69

[B28] Arno PS Levine C Memmott MM The economic value of informal caregiving Health Affairs 1999 14 182 188 10091447 10.1377/hlthaff.18.2.182

[B29] Peters LL Boter H Buskens E Slaets JP Measurement properties of the Groningen Frailty Indicator in home-dwelling and institutionalized elderly people J Am Med Dir Assoc 2012 14 6 546 551 22579590 10.1016/j.jamda.2012.04.007

[B30] Boot JM Knapen MJMH Handboek Nederlandse gezondheidszorg [Handbook Dutch Healthcare] 2005 Utrecht: Het Spectrum

[B31] Website National Care for the Elderly Program (NPO) http://www.nationaalprogrammaouderenzorg.nl/english/the-national-care-for-the-elderly-programme/

[B32] Lutomski JE Baars MAE Schalk BWM Boter H Buurman BM Den Elzen WPJ Jansen APD Kempen GIJM Steunenberg B Steyerberg EW Olde Rikkert MGM Melis RJF The development of The Older Persons and Informal Caregivers Survey Minimum DataSet (TOPICS-MDS): A large-scale data sharing initiative PLoS ONE 2013 14 12 e81673 24324716 10.1371/journal.pone.0081673 PMC3852259

[B33] Van der Zee KI Sanderman R Het meten van de algemene gezondheidstoestand met de RAND-36: Een handleiding [The assessment of general health status with the RAND-36] 2012 Groningen: Research Institute SHARE/University of Groningen, the Netherlands

[B34] Van den Berg B Spauwen P Measurement of informal care: An empirical study into the valid measurement of time spent on informal caregiving Health Economy 2006 14 5 447 460 10.1002/hec.1075 16389664

[B35] Hoefman RJ Van Exel NJA Rose JM Van De Wetering EJ Brouwer WB A discrete choice experiment to obtain a tariff for valuing informal care situations measured with the CarerQol instrument Medical Decision Making 2013 14 1 84 96 23771881 10.1177/0272989X13492013

[B36] Brouwer WB Van Exel NJA van den Berg B van den Bos GA Koopmanschap MA Process utility from providing informal care: The benefit of caring Health Policy 2005 14 1 85 99 16098415 10.1016/j.healthpol.2004.12.008

[B37] Van Exel NJA Scholte Op Reimer WJ Brouwer WBF Van Den Berg B Koopmanschap MA Van Den Bos GA Instruments for assessing the burden of informal caregiving for stroke patients in clinical practice: A comparison of CSI, CRA, SCQ and Self-Rated Burden Clin Rehabil 2004 14 2 203 214 15053130 10.1191/0269215504cr723oa

[B38] Cantril H The pattern of human concerns 1965 New Brunswick, NJ: Rutgers University Press

[B39] Deeken JF Taylor KL Mangan P Yabroff KR Ingham JM A review of self-report instruments developed to measure burden, needs, and quality of life of informal caregivers J Pain Symptom Manage 2003 14 4 922 953 14527761 10.1016/s0885-3924(03)00327-0

[B40] Schulz R Martire LM Family caregiving of persons with dementia: Prevalence, health effects, and support strategies Am J Geriatr Psychiatry 2004 14 3 240 249 15126224

[B41] Wolfs CAG Kessels A Severens JL Brouwer W De Vugt ME Verhey FRJ Dirksen CD Predictive factors for objective burden of informal care in people with dementia: A systematic review Gerontologist 2006 14 3 344 356 10.1097/WAD.0b013e31823a6108 22075941

[B42] Verhage F Intelligentie en leeftijd: Onderzoek bij Nederlanders van twaalf tot zevenenzeventig jaar 1964 Assen: Van Gorcum

[B43] Cohen J Statistical power analysis for the behavioral sciences 1988 2 Hillsdale, New Jersey: Lawrence Erlbaum Associates

[B44] Fields A Discovering statistics using SPSS 2009 3 London: Sage Publications Ltd.

[B45] Etters L Goodall D Harrison BE Caregiver burden among dementia patient caregivers: A review of the literature J Am Acad Nurse Pract 2008 14 8 423 428 18786017 10.1111/j.1745-7599.2008.00342.x

[B46] Sörensen S Duberstein P Gill D Pinquart M Dementia care: Mental health effects, intervention strategies, and clinical implications Lancet Neurology 2006 14 11 961 973 17052663 10.1016/S1474-4422(06)70599-3

[B47] Coon DW Evans B Empirically based treatments for family caregiver distress: What works and where do we go from here? Geriatr Nurs 2009 14 6 426 436 19998566 10.1016/j.gerinurse.2009.09.010

[B48] Pusey H Richards D A systematic review of the effectiveness of psychosocial interventions for carers of people with dementia Aging Ment Health 2001 14 2 107 119 11511058 10.1080/13607860120038302

[B49] Hasson H Systematic evaluation of implementation fidelity of complex interventions in health and social care Implementation Science 2010 14 1 67 20815872 10.1186/1748-5908-5-67 PMC2942793

[B50] Melis RJ Van Eijken MI Teerenstra S Van Achterberg T Parker SG Borm GF Van De Lisdonk EH Wensing M Olde Rickert MGM Multidimensional geriatric assessment: Back to the future. A Randomized study of a Multidisciplinary program to intervene on geriatric syndromes in vulnerable older people who live at home (Dutch EASYcare Study) Journals of Gerontology: Medical sciences 2008 14 3 283 90 10.1093/gerona/63.3.283 18375877

[B51] Callahan CM Boustani MA Unverzagt FW Austrom MG Damush TM Perkins AJ Fultz BA Hui SL Counsell SR Hendrie HC Effectiveness of collaborative care for older adults with Alzheimer disease in primary care: A randomized controlled trial J Am Med Assoc 2006 14 18 2148 2157 10.1001/jama.295.18.2148 16684985

[B52] Callahan CM Boustani M Sachs GA Hendrie HC Integrating care for older adults with cognitive impairment Curr Alzheimer Res 2009 14 4 368 374 19689236 10.2174/156720509788929228 PMC3319309

[B53] Bookwala J Schulz R Spousal similarity in subjective wellbeing: The Cardiovascular Health Study Psychol Aging 1996 14 4 582 590 9000291 10.1037//0882-7974.11.4.582

[B54] Tower RB Kasl SV Moritz DJ The influence of spouse cognitive impairment on respondents’ depressive symptoms: The moderating role of marital closeness Journal of Gerontology: Social Sciences 1997 14 5 S270 S278 10.1093/geronb/52b.5.s270 9310099

[B55] Sharpe L Butow P Smith C McConnell D Clarke S The relationship between available support, unmet needs and caregiver burden in patients with advanced cancer and their carers Psycho‒Oncology 2005 14 2 102 114 15386783 10.1002/pon.825

[B56] Bailis DS Segall A Chipperfield JG Two views of self-rated general health status Soc Sci Med 2003 14 2 203 217 12473308 10.1016/s0277-9536(02)00020-5

[B57] Pinquart M Sörensen S Helping caregivers of persons with dementia: Which interventions work and how large are their effects? Int Psychogeriatr 2006 14 4 577 595 16686964 10.1017/S1041610206003462

[B58] Campbell M Fitzpatrick R Haines A Kinmonth AL Sandercock P Spiegelhalter D Tyrer P Framework for design and evaluation of complex interventions to improve health Br Med J 2000 14 7262 694 696 10987780 10.1136/bmj.321.7262.694 PMC1118564

