# Supplementary material for: The effects of an integrated care intervention for the frail elderly on informal caregivers: a quasi-experimental study
Source: BMC Geriatr. 2014 May 1;14:58. doi: 10.1186/1471-2318-14-58 (PMC4021048; doi:10.1186/1471-2318-14-58)
Supplement: Additional file 1 — Description of file: English version of the questionnaire used in the current study (developed for the ‘Nationaal Programma Ouderenzorg’ (NPO) [National Care for the Elderly Program]. [file 1471-2318-14-58-S1.pdf]

QUESTIONNAIRE

Care giver

YOUR EXPERIENCES AS A CAREGIVER ARE VALUABLE

You are caring for your partner, a family member, a friend or a loved one. This list contains questions about what this care means for you as a caregiver. Your answers will be used in research aimed at improving elderly care and informal care. The more information there is about these types of care, the more they can be taken into consideration. For example when changing the facilities, regulations or legislation for care and wellbeing. Therefore, your experiences are also valuable for other caregivers. Your answers will be processed anonymously and your data will not be distributed further.
If you complete this questionnaire, this will not have any effect on the care that you or your loved one receives.

If you have any further questions, please call us on …

INSTRUCTIONS FOR THIS QUESTIONNAIRE

- Completing this questionnaire will take approximately twenty minutes.
- If you read 'your loved one', then the question refers to the person for whom you are a caregiver.
- If the questions are about you they will mention 'yourself'.
- Read each question through completely before answering.
- Then tick the answer that best fits your situation.
- Tick only one answer for each question.
- If you are allowed to tick more than one answer, then this will be mentioned for the relevant question.
- When you are done, please check that you have not forgotten any questions.

Your date of birth, gender and postal code

Please fill in your details below:

1 Your date of birth: ____ - ____ - ____

2 Your gender:

- ☐ Male
- ☐ Female

3 Postal code: | | | | | Only fill in the four figures here

Date of birth, gender and postal code of your loved one

Complete below:

4 Date of birth of your loved one: ____ - ____ - ____

5 Gender of your loved one:

- ☐ Male
- ☐ Female

6 Postal code: | | | | | Only fill in the four figures here

Relation to your loved one

7 What is your relationship with your loved one?

I am his/her…

- ☐ Husband / wife / life partner
- ☐ Sister (-in-law), brother (-in-law)
- ☐ Daughter (-in-law) / son (-in-law)
- ☐ Other, namely: ____________________

8 Do you live with your loved one?

- ☐ Yes
- ☐ No

Your health

The following questions are about your health. Please tick the answer that best fits your situation.

9 In general, would you say your health is

- ☐ Excellent
- ☐ Very good
- ☐ Good
- ☐ Fair
- ☐ Poor

10 Compared to one year ago, how would you rate your health in general now?

- ☐ Much better
- ☐ Somewhat better
- ☐ About the same
- ☐ Somewhat worse
- ☐ Much worse

Time spent caring for your loved one

The following questions are about the amount of time that you spend caring for your loved one. You will be asked to write down whether you have given your loved one assistance for various activities of daily life. With ‘assistance’, we mean that you have supported your loved one in the task or have taken over the task entirely.

Please fill in as accurately as possible the amount of time that you spend on all activities. For some caregivers it feels like they spend the whole day caring for their loved one. For example, because the care is very hard work. Even so, try to indicate as precisely as possible how much time you really spend caring for your loved one. There are questions further on in this questionnaire about how hard the care feels to you.

Complete the questions based on how the previous week has been. Choose another week if your time expenditure in the previous week has been very different to the average week, for example due to illness or vacation.

In the past week, have you had to help your loved one with the following due to his/her health problems …

11 household tasks such as preparing food and drinks, cleaning the house, washing, ironing and sewing clothing, shopping for groceries or odd jobs in the house or the garden?

☐ No

☐ Yes, namely ☐ hours per week

In the past week, have you had to help your loved one with the following due to his/her health problems …

12 personal care (dressing and undressing, washing, combing, shaving), going to the toilet, moving around the house, eating, drinking or administering medication?

☐ No

☐ Yes, namely ☐ hours per week

13 moving around outside the house, going on outings and visiting family or friends, contacts with healthcare (accompanying him/her for example to the general practitioner, the hospital, therapy), arranging assistance, devices and/or home modifications and organising financial and administrative matters?

☐ No

☐ Yes, namely ☐ hours per week

14 Does your loved one receive help from other caregivers or volunteers besides you?

☐ No

☐ Yes, namely ☐ hours per week

How difficult is the care for you

15 Indicate with a tick on the scale how hard you are finding it to care for your loved one. The closer to ‘0’, the less hard you find the care. The closer to ‘10’, the harder you find the care. (Also refer to the example below.)

Not difficult at all | **Far too difficult**

0 1 2 3 4 5 6 7 8 9 10

Do not like at all | **Like very much**

0 1 2 3 4 5 6 7 8 9 10

Example: How much do you like chocolate ice cream?

The following questions are about your situation as a caregiver. Place a tick next to the word that best describes your care situation: no/none, some or a lot.

16 I gain …

- ☐ no
- ☐ some
- ☐ a lot

… (of) satisfaction from performing my care tasks.

17 I have …

- ☐ no
- ☐ some
- ☐ a lot

… (of) problems with my loved one (for example, he/she is demanding, we have communication problems, he/she has started behaving differently).

18 I have …

- ☐ no
- ☐ some
- ☐ a lot

… (of) problems with my own mental health (for example stress, anxiety, despondency, concern about the future).

19 I have …

- ☐ no
- ☐ some
- ☐ a lot

… (of) problems with my own physical health (for example being sick more often, fatigue, physical over-exertion).

20 I have …

- ☐ no
- ☐ some
- ☐ a lot

… (of) problems combining my daily activities (for example work, household, education, family and free time) with my care tasks.

21 I have …

- ☐ no
- ☐ some
- ☐ a lot

… (of) financial problems concerning my care tasks.

22 I have …

- ☐ no
- ☐ some
- ☐ a lot

… (of) support (from family/friends/neighbours/acquaintances/volunteers) in performing my care tasks.

23 Indicate with a tick on the scale how happy you are feeling right now.

Completely unhappy | Perfectly happy

0 1 2 3 4 5 6 7 8 9 10

24 Imagine the following: you and your loved one may select someone yourselves who will take over all your care tasks. There are no extra costs involved and this person will provide all transferred care tasks at your loved one's home. Indicate by means of a tick on the scale how happy you would feel if this person would take over the care responsibilities from you.

Completely unhappy | Perfectly happy

0 1 2 3 4 5 6 7 8 9 10

Quality of life for yourself

The following questions are about your ‘quality of life’. This refers to what you think about your life. For example, whether you are satisfied with your life, whether you have enjoyment in your life and whether your life gives you satisfaction. Please tick the box of the answer that best fits your situation.

25 How is your quality of life in general?

- ☐ Excellent
- ☐ Very good
- ☐ Good
- ☐ Reasonable
- ☐ Poor

26 Which report mark would you give your life at this moment?

Report mark: ☐ Enter a figure between 0 and 10 here

27 How is your quality of life in general, in comparison to one year ago?

- ☐ Much better
- ☐ Slightly better
- ☐ About the same
- ☐ Slightly worse
- ☐ Much worse

Conclusion

If you have any comments, please write them down in the space below:

This is the end of the questionnaire.

Thank you very much for completing the questionnaire!!!
