# Supplementary material for: The effects of an integrated care intervention for the frail elderly on informal caregivers: a quasi-experimental study
Source: BMC Geriatr. 2014 May 1;14:58. doi: 10.1186/1471-2318-14-58 (PMC4021048; doi:10.1186/1471-2318-14-58)
Supplement: Additional file 2 — Description of file: Table showing the contribution of each model to the R2 and their significance. [file 1471-2318-14-58-S2.docx]

Additional File 2. Contribution of Each Regression Model to the *R2* and their Significance

| Outcome Variables | Model 1  (T0) | | Model 2  (+control variables) | | Model 3  (+intervention) | |
| --- | --- | --- | --- | --- | --- | --- |
|  | % R2 | Sign. | % R2 | Sign. | % R2 | Sign. |
| Perceived health | 48.1 | 0.000 | 51.0 | 0.051 | 51.3 | 0.212 |
| Perceived change in health | 4.6 | 0.007 | 23.4 | 0.000 | 22.9 | 0.587 |
| CarerQoL sum | 39.7 | 0.000 | 43.2 | 0.053 | 44.5 | 0.053 |
| CarerQoL-VAS | 33.2 | 0.000 | 32.1 | 0.670 | 32.4 | 0.195 |
| Process Utility | 34.3 | 0.000 | 37.4 | 0.082 | 37.1 | 0.568 |
| SRB | 15.0 | 0.000 | 19.5 | 0.063 | 19.1 | 0.524 |
| % household tasks (log) | 23.2 | 0.000 | 33.5 | 0.001 | 38.3 | 0.001 |
| % personal care tasks (log) | 33.5 | 0.000 | 45.5 | 0.000 | 46.0 | 0.000 |
| % instrumental care tasks (log) | 18.7 | 0.000 | 34.4 | 0.000 | 34.6 | 0.000 |
| % additional caregivers (log) | 35.9 | 0.000 | 45.0 | 0.000 | 45.1 | 0.000 |
| Hours household tasks | 31.8 | 0.000 | 42.8 | 0.000 | 42.3 | 0.777 |
| Hours personal care tasks | 43.8 | 0.000 | 44.8 | 0.247 | 44.4 | 0.895 |
| Hours instrumental care tasks | - | NS | - | NS | - | NS |
| Total hours | 47.4 | 0.000 | 49.7 | 0.112 | 49.8 | 0.295 |
| Hours other caregivers | 29.8 | 0.000 | 35.0 | 0.033 | 35.0 | 0.313 |
| Total hours + other caregivers | 43.8 | 0.000 | 44.3 | 0.364 | 44.9 | 0.150 |
| Quality of life | 21.5 | 0.000 | 32.6 | 0.001 | 32.2 | 0.533 |
| Change in quality of life | - | NS | 4.4 | 0.065 | - | NS |
| Rating of quality of life | 18.2 | 0.000 | 23.5 | 0.035 | 23.3 | 0.453 |

*Note:* % R2 and significance = adjusted R2 and F-change for linear regression; Nagelkerke’s R2 and model chi-square for logistic regression. NS = non-significant model.
